# Effect of Different Terpene-Containing Essential Oils on the Proliferation of *Echinococcus granulosus* Larval Cells

**DOI:** 10.1155/2014/746931

**Published:** 2014-09-28

**Authors:** Clara María Albani, Guillermo María Denegri, María Celina Elissondo

**Affiliations:** ^1^Laboratorio de Zoonosis Parasitarias, Departamento de Biología, Facultad de Ciencias Exactas y Naturales, Universidad Nacional de Mar del Plata (UNMdP), Funes 3350, B7602AYL Mar del Plata, Argentina; ^2^Consejo Nacional de Investigaciones Científicas y Técnicas (CONICET), Buenos Aires, Argentina

## Abstract

Human cystic echinococcosis remains a major public health problem on several countries and the treatment strategies are not solved. The aim of the present work was to determine the in vitro effect of thymol and* Mentha piperita*,* M. pulegium, *and* Rosmarinus officinalis *essential oils on the proliferation of* E. granulosus *larval cells. Isolated cells and cellular aggregates were obtained from hydatid cyst's germinal layer and exposed to 1, 5, and 10 *μ*g/ml of thymol and the different essential oils for 7 days. Drug effect was evaluated using test viability and scanning electron microscopy. Control cell culture viability was 2.1 x 10^6^ (100%) after 7 days of incubation. At day 7, thymol 5 *μ*g/ml caused a reduction in cell viability of 63% and the essential oils of* M. piperita *10 *μ*g/ml,* M. pulegium *10 *μ*g/ml, and* R. officinalis *10 *μ*g/ml produced a reduction in the viability of 77, 82, and 71%, respectively. Moreover essential oils caused reduction in cell number, collapsed cells, and loss of normal tridimensional composition of the aggregates. Due to the inhibitory effect caused by essential oils on* E. granulosus *cells we suggested that it would be an effective means for suppression of larval growth.

## 1. Introduction

Cystic echinococcosis (CE) is a zoonotic disease caused by the larval stage of the parasite* Echinococcus granulosus* which has a worldwide distribution and important medical and economic impact [1]. Metacestode or hydatid cyst is composed of three layers, an inner germinal layer which contains several cell types including undifferentiated cells, as well as muscle and tegumentary cells. This layer is supported externally by a noncellular laminated layer, which is surrounded by a host-produced adventitial layer [2].

Traditionally, surgery constitutes the most used alternative of CE treatment since it has the potential to remove the cysts and lead to complete cure [3]. However, in the case of inoperable patients or to accompany surgery, chemotherapy results in a better option. To date there is no alternative treatment with 100% efficacy. Therefore, a strong impetus for researchers to develop alternative treatment methods, such as the use of traditional medicinal plants, has recently been promoted [4].

At present, the methods used for drug screening against cystic echinococcosis include the incubation of protoscoleces or murine cysts with the substances to test. Using this methodology to the present several encouraging findings have been reported [5–9]. Although it has been suggested that essential oils are presented as a valuable therapeutic option against a large number of diseases [10, 11], to date there are just a few works that study the role of essential oils specifically against parasitic helminths [12–14]. Recently the* in vitro* protoscolicidal effect of essential oil of* Rosmarinus officinalis* (rosemary), thymol (principal component of essential oils extracted from* Origanum vulgare* and* Thymus vulgare*), essential oil and extract of* Pistacia khinjuk,* and essential oil of* Mentha* spp. has been demonstrated by several authors [4, 5, 7, 15].

*In vitro* culture systems are very useful to characterize candidate compounds with regard to their mechanism of action [16]. Innovatively, we propose the use of an* E. granulosus* cell culture as a tool to carry out mass screening for anthelmintic compounds.

The aim of the present work was to determine the* in vitro* effect of different terpene-containing essential oils on the proliferation of* E. granulosus* larval cells.

## 2. Materials and Methods

### 2.1. Plant Material

The essential oils were kindly provided by Dr. Liesel Gende and Dr. Martín Eguaras (Laboratorio de Artrópodos, Facultad de Ciencias Exactas y Naturales, Universidad Nacional de Mar del Plata). The* R. officinalis* essential oil was extracted as reported by [5] and the* M. piperita* and* M. pulegium* as reported by [7].

### 2.2. Cell Culture and Drug Treatment

*E. granulosus* cell culture was obtained using previously reported methods [17]. In brief cells were cultured at 37°C in medium 199 (Gibco BRL) supplemented with 10% FBS, 10% hydatid fluid, reducing agents (5 × 10^−5 ^M 2-mercaptoethanol and 100 *μ*M L-cysteine), 2 mM L-glutamine (Bio-Rad, USA), 4 mg mL^−1^ glucose (Sigma, USA), 1 mM sodium pyruvate (Sigma, USA), and antibiotics (penicillin, streptomycin, and gentamicin 100 *μ*g mL^−1^).* E. granulosus* cells were cultivated for at least 4 weeks. Some cultures were subcultured once a week and others were maintained without subculture to promote the formation of cellular aggregates growing in suspension. Thymol (Sigma) was dissolved in dimethyl sulphoxide (DMSO) at a drug concentration of 10 mg/mL. Essential oils of* M. piperita*,* M. pulegium,* and* R. officinalis* were dissolved in 10 mL of distilled water and were emulsified with propylene glycol (PG) to 5% v/v. Albendazole (ABZ) (Sigma-Aldrich) was used as reference drug and the solution was prepared by dissolution of 10 mg of pure standard drug in 1 mL of DMSO. Then, thymol, ABZ, or each essential oil was added to the medium resulting in final concentrations of 10, 5, and 1 *μ*g/mL. Cells incubated in culture medium containing 10 *μ*L DMSO or PG served as controls. Each experiment was repeated three times.

### 2.3. Growth Inhibitory Assay on Isolated Cells

*E. granulosus* cells were seeded into 24-well microplates to achieve an approximate density of 5 × 10^5^ cells well^−1^ in 1 mL medium. For this experiment cell cultures after 24 h of subculture were used. Thymol or the different essential oils were added in serial concentrations and cultures were incubated for 7 days. At days 0, 2, 5, and 7 viability was assessed by trypan blue dye (Sigma) exclusion using a hemocytometer. During the experiments, cultures were followed microscopically to determine the appearance of morphological alterations.

### 2.4. Drug Effect on Isolated Cells and Cell Aggregates

For this experiment cell cultures after 24 h of subculture or four-month-old cultures containing big amount of cell aggregates were used. At days 2, 5, and 7 of treatment, samples were harvested (aggregates were recovered in 1.5 mL plastic tubes and individual cells were treated for the whole procedure attached in the slides), fixed with 2.5% (v/v) glutaraldehyde in 0.1% (v/v) sodium cacodylate buffer for 48 h at 4°C, and then washed several times in cacodylate buffer. The specimens were later dehydrated by sequential incubations in increasing concentrations of ethanol (50% 10 min, 70% 10 min, 80% 10 min, 90% 10 min, 95% 10 min, 100% 10 min twice) and finally immersed in hexamethyldisilazane for 18 h. They were sputter-coated with gold and inspected on a JEOL JSM-6460 LV scanning electron microscope (Japan) at 15 kV.

### 2.5. Statistical Analysis

In order to test the experimental hypothesis generalized least squares (GLS) method was applied [18]. Analyses were performed using the statistical program “R” (2011) [19] and the statistical package “NLME” [20].

## 3. Results

**Figure 1:**
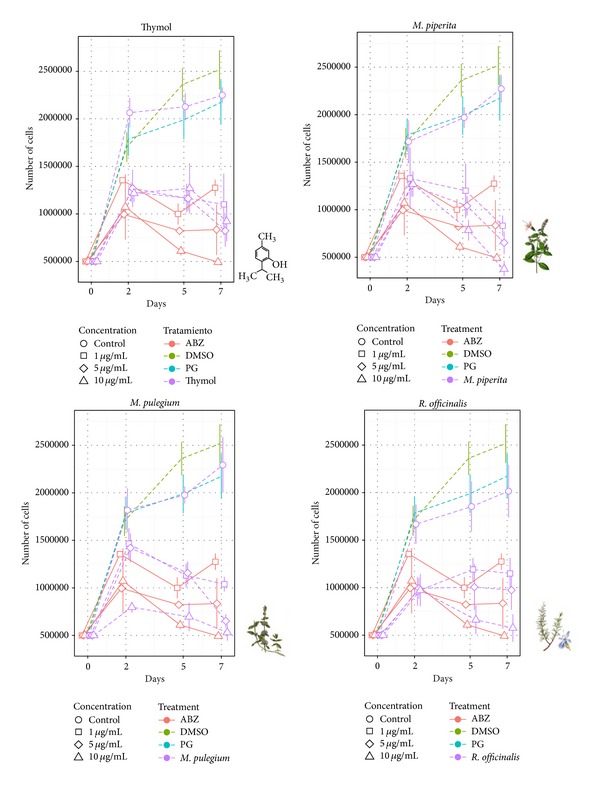
Survival of* E. granulosus* larval cells after treatment with* M. pulegium*,* M. piperita*,* R. officinalis *essential oils and thymol. Each point represents the mean percentage of vital cells from three different experiments ± SD.

Cell culture survival after exposure to different concentrations of thymol and the essential oils is shown in Figure 1. Control treatment showed always an increase in cell number reaching an average of 2.1 × 10^6^ total cells at day 7. No differences were found when medium containing PG or DMSO was used as control (Figure 1).

Thymol produced a diminishing in cells number only in a time-dependent manner. The highest anthelmintic effect of thymol was obtained with 5 *μ*g/mL showing a reduction of 63% in cell viability at day 7. Nevertheless no differences between the different concentrations of thymol were detected. The essential oils of* M. piperita* and* M. pulegium *produced dose- and time-dependent effects only towards the end of the experiment reaching a viability reduction of 77 and 82%, respectively, with 10 *μ*g/mL concentration at day 7. Theessential oil of* R. officinalis *showed dose- and time-dependent effects showing a reduction in the cell viability of 71% at day 7 (10 *μ*g/mL). Treatment with thymol or the different essential oils not only leads to the arrest of cell division but also provoked a considerable reduction in the number of cells from day 2 after incubation. On the other hand, big amount of cellular debris could be detected during cell counts especially using the highest concentrations of phytotherapics. ABZ was used as reference drug showing no difference from the treatments assayed.

**Figure 2:**
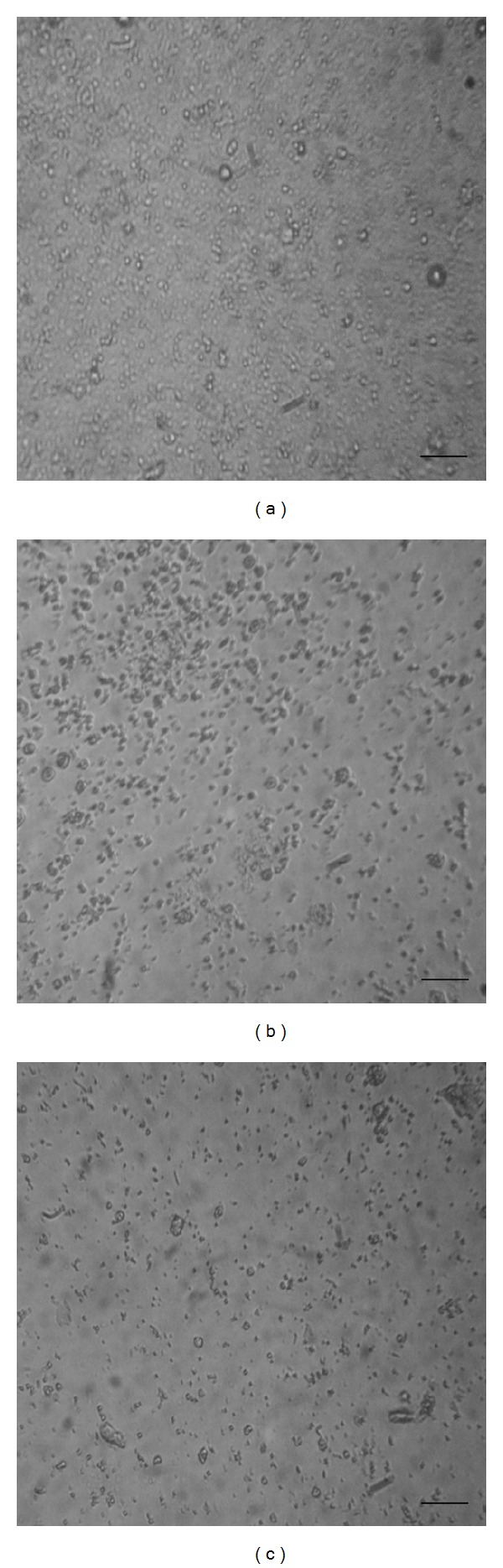
Optical microscopy of* E. granulosus* larval cells treated for 7 days with* R. officinalis* essential oil. (a) Control. (b) Cells treated with 5 *μ*g/mL. (c) Cells treated with 10 *μ*g/mL. Bar = 40 *μ*m.

**Figure 3:**
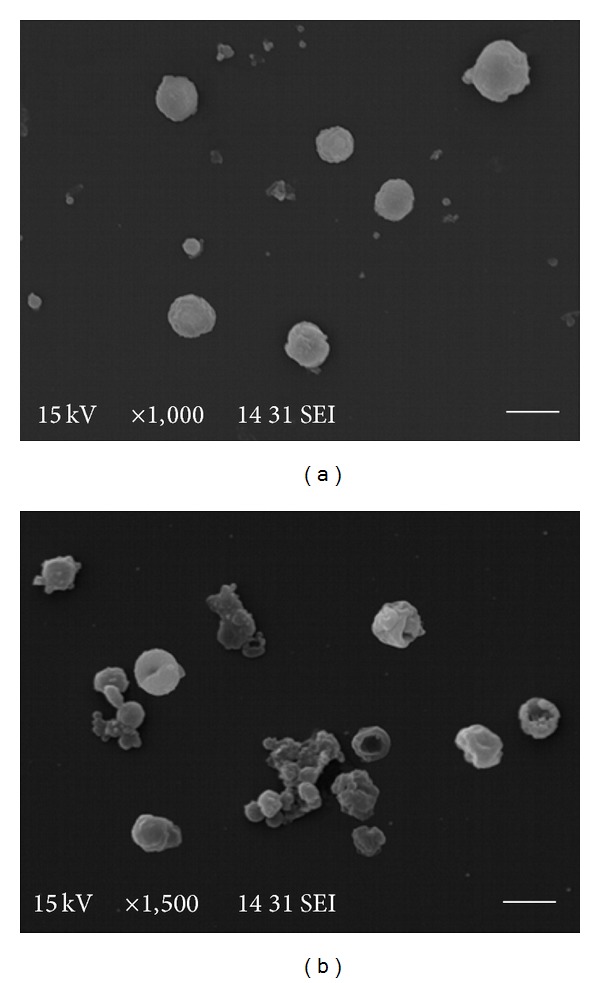
Scanning electron microscopy of* E. granulosus *isolated larval cells incubated* in vitro* with 10 *μ*g/mL of thymol during 7 days. (a) Control. (b) Cells treated with thymol. Bar = 5 *μ*m.

The effect of thymol,* Mentha* spp., and* R. officinalis* essential oils was also monitored by optical microscopy showing not only a reduction in the cell number matching with the results obtained in the viability test (Figure 2) but also many dead cells and cellular debris. Moreover, studies by SEM revealed that ultrastructural damage was produced in drug-treated cells. There was a correlation between the intensity of damage and the concentration of the essential oil or compound assayed. Cells treated with thymol 10 *μ*g/mL at day 7 showed morphological alteration as loss of turgidity, cellular contraction, and reduction in the cell number (Figure 3).

**Figure 4:**
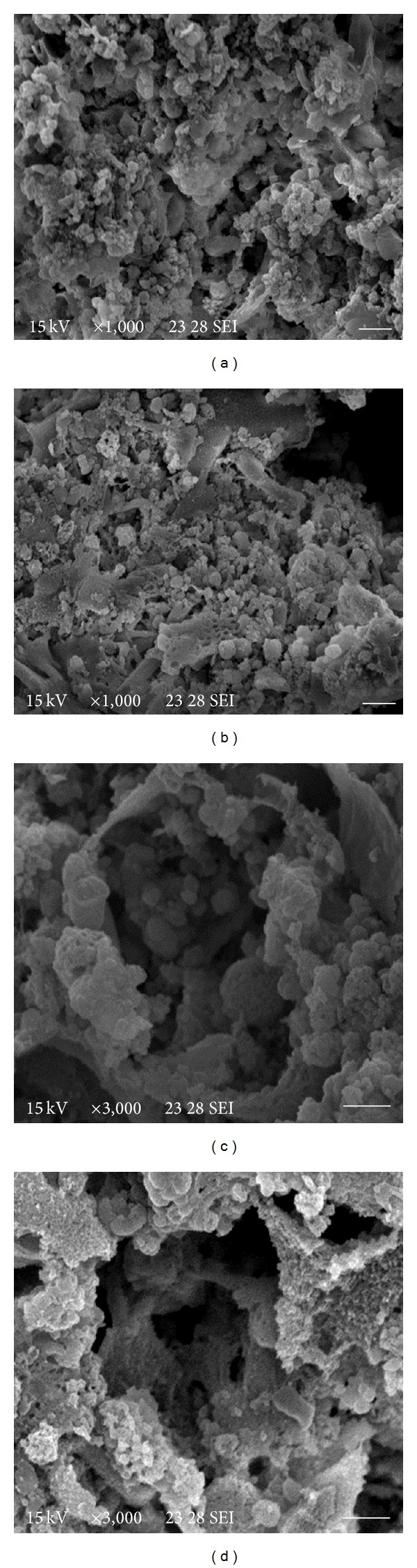
Scanning electron microscopy of* E. granulosus *larval cell aggregates incubated* in vitro* with different essential oils. (a) and (c) Control cell aggregates exhibiting the typical tridimensional organization. (b) Cell aggregates incubated with 10 *μ*g/mL of* R. officinalis* essential oil for 7 days. (d) Cell aggregates incubated with 10 *μ*g/mL of* M. piperita* essential oil for 7 days. Note the loss of the typical multicellular structure. Bar = (a) and (b) 10 *μ*m, (c) and (d) 5 *μ*m.

On the other hand, structural damage induced on the cellular aggregates by the drugs was also observed.  Figure 4(a) shows untreated cellular aggregates exhibiting the typical tridimensional composition as a rough surface with big amount of cells and the presence of cavities. After 7-day postincubation with thymol or the essential oils, severe alterations on the aggregates surface were observed.  Figure 4(b) shows the effect of* R. officinalis *as a marked reduction in cell number, presence of cellular debris and collapsed cells. The cavities appeared as empty spaces surrounded by a wall of 2-3 cells of thickness (Figure 4(c)). The effect of the phytotherapics on the cavities which showed loss of the typical multicellular structure was also observed (Figure 4(d)).

The statistical analysis (GLS) revealed that the studied factors (concentration, exposition time, and treatment) were relevant to explain the cell number variation.

## 4. Discussion

Essential oils and plant extracts have been used for thousands of years especially for food preservation, pharmaceuticals, alternative medicine, and natural therapies [21]. Recently, herbal medicines have increasingly been used to treat many diseases including several infections [11] and also for several volatile oils (monoterpenoids and sesquiterpenoids), antimicrobial as well as anticancer activity has been reported [22].

Several studies have demonstrated the* in vitro* effect of various essential oils against helminths [23–26]. It was also demonstrated that* Mentha* spp. and* R. officinalis* essential oils and the main component of the essential oils of* T. vulgaris* and* O. vulgare*, thymol, have a marked* in vitro* anthelmintic activity [4, 5, 7].

This paper describes for the first time the* in vitro* effect of different terpene-containing essential oils on the proliferation of* E. granulosus* larval cells. The anthelmintic activity of thymol and the essential oils of* M. piperita*,* M. pulegium,* and* R. officinalis* was demonstrated. Treatments always showed differences with the control condition; however unlike treatment with essential oils, thymol showed no differences between the concentrations employed. It could be due to the lower concentrations used. In previous studies the antiparasitic effect of essential oils on trypanosomatids was studied and the use of concentrations between 20 and 150 *μ*g/mL was reported [27].

A decrease in* E. granulosus* cell number proportional to the exposure time was observed.* M. pulegium* (10 *μ*g/mL) produced the fastest effect with a reduction in cell viability of 56% at day 2. On the other hand the highest anthelmintic effect was observed with* M. piperita* (10 *μ*g/mL) which caused a reduction in cell viability of 82% at day 7.

These results are consistent with those reported by [7], who observed a marked protoscolicidal effect using* M. pulegium* and* M. piperita* essential oils, even though the effect obtained with cells was faster. It could be explained by the fact that protoscoleces are multicellular organisms surrounded by tegument in contrast with naked cells that were more exposed to the action of the drugs. Due to the fact that these cells are the same who compose the germinal layer this result would be extrapolated to the drug effect on the hydatid cyst.

The evaluated phytotherapics caused structural changes in both* E. granulosus* individual cells and cell aggregates, producing mainly shrinkage, loss of cell turgidity, and disappearance of the normal multicellular organization of aggregates. This result agrees with those observed in previous studies after treatment of hydatid cyst germinal layer with different drugs [7, 28].

Although to the present little is known about the mode action of essential oils, it is believed that it involves different targets and mechanisms in the different organisms due to the fact that they contain great variety of components [29]. The main compounds of the essential oils used in this work were piperitone oxide (63.6%) for* M. pulegium*, isomenthol (36.08%) for* M. piperita,* and beta-myrcene (24.97%) for* R. officinalis*. It has been reported that piperitone oxide showed an inhibitory effect against* Staphylococcus aureus* [30],* Aspergillus flavus* [31], and Enterobacteriaceae [32]. On the other hand, the antibacterial effect of menthol (isomenthol's isomer) was demonstrated by several authors [33, 34]. Moreover the antioxidant and antibacterial properties of beta-myrcene were proved [35].

In comparison with the low concentrations used in our study, for thymol LD50 value of 400 *μ*g/mL using U-937 human promonocytic cells has been reported [36]. In the case of the essential oils of* M. piperita*,* M. pulegium* and* R. officinalis *LD50 values of 1612.45 mg/kg [37], 4200 mg/kg [38] and >2000 mg/kg [39], were reported respectively employing rodent models. These data give evidence that the phytotherapics used here were more selective against* E. granulosus* larval cells compared with other animal models.

The* E. granulosus* cell culture model was suitable for the screening of various essential oils and components with the aim of finding new anthelmintic drugs. Moreover it allowed analyzing the drug effect on cell proliferation which is not possible using protoscoleces, microcysts, or murine cysts. Due to the inhibitory effect observed on* E. granulosus* cells which was comparable with the results obtained on germinal layer, we suggested that it would be an effective means for suppression of larval growth.

In future work, we plan to evaluate the anthelmintic activity of essential oils components separately or combined to assess the occurrence of synergistic or antagonistic interactions between them. Such studies could identify and quantify the responsibility of each component in the anthelmintic activity. The use of the individual components would provide greater predictability and less likelihood of side effects.
